# The Tobit-Unscented-Kalman-Filter-Based Attitude Estimation Algorithm Using the Star Sensor and Inertial Gyro Combination

**DOI:** 10.3390/mi14061243

**Published:** 2023-06-13

**Authors:** Xinmei Wang, Hui Zhang, Xiaodong Gao, Rujin Zhao

**Affiliations:** 1 National Key Laboratory of Optical Field Manipulation Science and Technology, Chinese Academy of Sciences, Chengdu 610209, China; wangxinm1006@163.com (X.W.);; 2 Key Laboratory of Science and Technology on Space Optoelectronic Precision Measurement, Chinese Academy of Sciences, Chengdu 610209, China; 3 Institute of Optics and Electronics Chinese Academy of Sciences, Chengdu 610209, China; 4 University of Chinese Academy of Sciences, Beijing 100049, China

**Keywords:** integrated navigation, Tobit model, TUKF

## Abstract

For the orbit operation of spacecraft, due to environmental factors, a star sensor installed on the spacecraft must have data censoring, which greatly reduces the attitude determination ability of the traditional combined-attitude-determination algorithm. To address this problem, this paper proposes an algorithm for high-precision attitude estimation based on a Tobit unscented Kalman filter. This is on the basis of establishing the nonlinear state equation of the integrated star sensor and gyroscope navigation system. The measurement update process of the unscented Kalman filter is improved. The Tobit model is used to describe the gyroscope drift when the star sensor fails. The latent measurement values are calculated using the probability statistics, and the measurement error covariance expression is derived. The proposed design is verified via computer simulations. When the star sensor fails for 15 min, the accuracy of the Tobit unscented Kalman filter based on the Tobit model is improved by approximately 90% compared to the unscented Kalman filter. Based on the results, the proposed filter can effectively estimate the error caused by the gyro drift, and the method is effective and feasible, provided there is theoretical support for the engineering practice.

## 1. Introduction

In engineering applications, sensor measurement has often been saturated, or there has been no output due to disturbance and occlusion, which affects the available measurement range of a detector, causing censoring failure. If a censored measurement result is treated as a normal measurement value or a truncated measurement result is completely discarded, the estimation result may differ significantly from the normal value of a censored area. In a satellite attitude-determination system, due to the influence of the space environment, a star sensor can fail, and the output measurement result can be lost [1]. In the process of the integrated navigation of a star sensor and an inertial gyro, when the data of a star sensor are censored, the inertial navigation information is generally used to determine a satellite’s attitude. However, due to the accumulation of the inertial navigation error over time, the resulting satellite attitude error is large [2]. In addition, censoring the star sensor output data can significantly reduce the attitude determination ability of the common attitude-determination algorithms, such as the extended Kalman filter (EKF), particle filter (PF), and unscented Kalman filter (UKF) [3,4,5,6]. In [7], the ANFIS theory is introduced to solve the combined navigation problem of a star sensor being in a transient navigation failure state, which has a large amount of reserved information and a complex calculation. In [8], a set of Bernoulli distribution random variables are used to model the delay sensor, and a piecewise analysis is proposed to modify the EKF, but it is not real time. In [9], a modified Square Root Unscented Kalman Filter (MSRUKF) is developed and used to estimate and control the angular velocity and attitude of a rigid gyro-less satellite in the presence of external disturbances and sensor faults. This method has a large amount of retained information and complex calculations, as well as the use of additional components, which increases costs. In [10], Soken and Hajiyev studied the robust UKF by introducing a scaling factor to adjust the gain of the Kalman filter, thereby weakening the impact of the abnormal measurements on filtering solutions. However, the proportion factor must be determined based on experience, which may lead to poor or biased filtering solutions. In this paper, to continue the real-time functioning and simplicity of the Kalman filter, a Tobit model is introduced to describe the measurement state.

The Tobit model was first proposed by economist James Tobin in 1958 to accurately simulate household expenditure [11,12]. The subsequent work proposed the many off-line estimation methods of the Tobit model [13,14,15,16,17,18,19]. Bethany Allik calculated the estimation deviation of the standard Kalman filter using the Tobit model [13,14,15]. It was assumed that there was no Jacobian matrix of the censored data of the EKF, and the steep discontinuity of the censored PF data caused a massive computational burden; the UKF had low computational overhead but was not robust enough on the censored interval. In [16], the censored and uncensored data were clearly separated and treated differently. The proposed solution represented an iterative maximum likelihood solution, which required using the measured historical data. In addition, it had a heavy computational burden and high memory requirements, thus reducing the Kalman filter performance. The Kalman filter introduced in [17,18,19] treated the censored measurement data as intermittent measurement data. This method provided a minimum state error variance and assumed that the missing measurement was not related to the state value. It can obtain a reasonable estimation result by using a complex state transition model. However, when a conserved measurement result is related to the state value, a biased estimation will be caused. In recent years, the Tobit model has been used more and more widely. The trajectory roll rate is estimated using the Tobit Kalman filter (TKF) with the aid of the enhancement technique and orthogonal projection principle [20,21]. The TKF is used to estimate the hidden state vectors in human skeletal tracking [22]. The Adaptive Tobit Kalman Filter (ATKF) is tested in an IoT positioning application and improve the estimation and tracking of variables with censored sensor data [23]. The Tobit unscented Kalman Filter (TUKF) is used to solve the target tracking problem with limited detection distance [24]. The Tobit model generally handles state quantities of one dimension [25]. However, in the problem of satellite-integrated navigation attitude determination, the state quantities are multidimensional. In summary, this paper presents the TUKF algorithm based on the Tobit model to improve the high accuracy attitude determination capability of the satellite platform when the star sensor fails.

This article proposes a TUKF that combines the Tobit model with the UKF, overcoming the impact of truncation measurement on attitude determination performance. This method uses the attitude kinematics equation and the gyro measurement equation to rewrite the state equation, and further describes the state estimation of the attitude error. Based on this, the statistical information of the truncated measurement is used to approximate the probability density function (PDF), and the error covariance matrix is calculated to make the estimation error closer to the true value and further improve the attitude determination accuracy. This method overcomes the limitations of the classical UKF by estimating and adjusting system noise statistics online. The simulation experiments were conducted in the integrated navigation of star sensors and gyroscopes with failed star sensors to evaluate the performance of the TUKF.

The remainder of this paper is organized as follows: Section 2 presents the Tobit model and the measurement model of a star sensor and a gyroscope integrated navigation. Section 3 outlines the TKF algorithm. Without data censoring, it shows the equivalence of TKF and the standard Kalman filter. Additionally, the TUKF is proposed using the covariance and expectation of the system state estimation. Additionally, the simulation results are presented in Section 4. The conclusions are drawn in Section 5.

## 2. Problem Formulation

### 2.1. Tobit Model

The Tobit model is a mathematical model describing data truncation proposed by James Tobin in [11]. The continuous variable is sometimes truncated or censored. If the intercepted value is directly used, it will lead to inconsistent estimators. Therefore, the probability density of the mixed distribution needs to be derived before estimation.

When there are censored data of a measurement model, the state equation and measurement equation of the model can be expressed as follow [13]:

(1) $$\begin{array}{c} x_{k}=Ax_{k-1}+\omega_{k-1}\\ y_{k}^{*}=Cx_{k}+v_{k}\\ y_{k}=\left\{\begin{array}{cc} y_{k}^{*} & y_{k}^{*}\leq \tau \\ \tau & y_{k}^{*}>\tau \end{array}\right. \end{array},$$

where $x_{k}$ is the state vector; A is the state transition matrix; C is the measurement matrix; $\omega_{k}\;\mathrm{and}\;v_{k}$ are mutually independent Gaussian white noises, having the covariances of Q and R, respectively; σ is the standard deviation; τ is the censored value of the Tobit model; $y_{k}$ is the estimated measurement; and $y_{k}^{*}$ is the latent measurement.

The Bernoulli random variable μ is introduced to simulate the measurement in the censored and uncensored areas. The measurement value at $t_{\tau }$ time is $\mu_{k}\left(l\right)=1\;(l=1,2\ldots m)$, after censoring for time $t_{\tau }$, $\mu_{k}\left(l\right)=0$. The measurement model of the Bernoulli variable is expressed as follows:

(2) $$\mu_{k}\left(l\right)=\left\{\begin{array}{c} 1,t\leq t_{\tau }\\ 0,t>t_{\tau } \end{array}.\right.$$

At a given time point, the probability of the *l*th measured element is the expected value $E\left(\mu_{k}\left(l\right)\right),where\;\mu_{k}\left(l\right)$ is a $R_{m*m}$ diagonal matrix when the matrix representation is used. The measurement equation is provided by:

(3) $$y_{k}=\mu_{k}y_{k}^{*}+\left(I_{m*m}-\mu_{k}\right)\tau .$$

In the Tobit model, it is necessary to reconsider the distribution of $y_{k}$. The distribution of the censored data with functional noise distribution is provided by:

(4) $$f\left(y_{k}\left|x_{k}\right.\right)=\frac{1}{\sigma }\phi \left(\frac{y_{k}-Cx_{k}}{\sigma }\right)u\left(y_{k}-\tau \right)+\delta \left(\tau -y_{k}\right)\Phi \left(\frac{Cx_{k}-\tau }{\sigma }\right),$$

where

(5) $$\phi \left(\frac{y_{k}-Cx_{k}}{\sigma }\right)=\frac{1}{\sqrt{2\pi }}e^{-\frac{{\left(y_{k}-Cx_{k}\right)}^{2}}{2\sigma^{2}}},$$

(6) $$\Phi \left(\frac{y_{k}-Cx_{k}}{\sigma }\right)=\int_{-\infty }^{y_{k}}\frac{1}{\sqrt{2\pi }}e^{-\frac{{\left(z-Cx_{k}\right)}^{2}}{2\sigma^{2}}}dz,$$

where ϕ and Φ are the PDF and the cumulative distribution function (CDF) of the standard normal distribution, respectively; *δ* is the unit impulse function; *u*(*α*) is the unit step function; and when *α* ≥ 0, *u*(*α*) = 1, but when *α* < 0, then *u*(*α*) = 0.

For the PDF of the actual measured data, the mathematical expectation and covariance are as follows [11]:

(7) $$E\left(y_{k}\left|x_{k}\right.,\sigma \right)=\Phi \left(\Lambda_{k}^{l}\right)\left(Cx_{k}-\sigma \lambda \left(\Lambda_{k}^{l}\right)\right)+\left(1-\Phi \left(\Lambda_{k}^{l}\right)\right)\tau ,$$

(8) $$Var\left(y_{k}\left|x_{k}\right.,\sigma \right)=\Phi \left(\Lambda_{k}^{l}\right)\sigma \left(1-\Psi \left(-\Lambda_{k}^{l}\right)\right),$$

where $\Lambda_{k}^{l}=\frac{\tau -Cx_{k}}{\sigma },\lambda \left(\Lambda_{k}^{l}\right)=\frac{\phi \left(\Lambda_{k}^{l}\right)}{\Phi \left(\Lambda_{k}^{l}\right)},\Psi \left(-\Lambda_{k}^{l}\right)=\lambda \left(\Lambda_{k}^{l}\right)\left(\lambda \left(\Lambda_{k}^{l}\right)-\left({-\Lambda }_{k}^{l}\right)\right)$.

### 2.2. Attitude Measurement Model Based on Star Sensor and Gyro

In the integrated navigation process of a star sensor and a gyro, when the star sensor fails, the attitude determination process of the spacecraft is transformed into the attitude estimation process based on the gyro’s data. Since the gyro error accumulates over time, a gyro drift compensation algorithm is used to correct the estimated attitude value, and the spacecraft attitude is obtained under the condition of missing star sensor data in a short time.

The measurement data of a gyro are provided by [26]:

(9) $$\omega \left(t\right)=u\left(t\right)+b\left(t\right)+n_{1}\left(t\right),$$

(10) $$b\left(t\right)=b_{0}+d\left(t\right),$$

where ${\omega =[\begin{array}{ccc} \omega_{x} & \omega_{y} & \omega_{z} \end{array}]}^{T}$ is the output angular velocity of a gyroscope; ${u=[\begin{array}{ccc} u_{x} & u_{y} & u_{z} \end{array}]}^{T}$ is the true angular velocity of the gyroscope relative to the inertial system; b is the gyro-related drift value; $b_{0}$ is the constant gyro drift value, $b_{0}\in R^{3*1};d\left(t\right)$ is random drift, and $d\in R^{3*1}$; and $n_{1}$ is the Gaussian white noise with the zero mean.

The random drift of the gyro satisfies the following condition:

(11) $$\dot{d}=n_{d},$$

where $n_{d}$ is white noise.

According to the attitude dynamics and kinematics of a satellite [27], the state equation of an attitude determination system is provided by:

(12) $$\dot{q}\left(t\right)=\frac{1}{2}\Omega \left(\omega^{b}\right)q\left(t\right)=\frac{1}{2}\psi \left(q\left(t\right)\right)\omega^{b0},$$

where $q={\left[q_{4}\;q_{1}\;q_{2}\;q_{3}\right]}^{T},q_{4}$ is a scalar value; $\overset{-}{q}=[q_{1}\;q_{2}\;q_{3}]$; and $\omega^{b}{=[\begin{array}{ccc} \omega_{x}^{b} & \omega_{y}^{b} & \omega_{z}^{b} \end{array}]}^{T}$ are the attitude quaternion and attitude angular velocity of the satellite body coordinate system (B) relative to the orbital coordinate system ($O_{x_{0}y_{0}z_{0}}$), respectively; $\omega^{b0}={\left[0\;\omega_{x}^{b}\;\omega_{y}^{b}\;\omega_{y}^{b}\right]}^{T}$ is the extended angular velocity; and $\Omega \left(\omega^{b}\right)=\left[\begin{array}{cc} \begin{array}{cc} 0 & {-\omega }_{x}^{b}\\ \omega_{x}^{b} & 0 \end{array} & \begin{array}{cc} {-\omega }_{y}^{b} & {-\omega }_{z}^{b}\\ \omega_{z}^{b} & {-\omega }_{y}^{b} \end{array}\\ \begin{array}{cc} \omega_{y}^{b} & {-\omega }_{z}^{b}\\ \omega_{z}^{b} & \omega_{y}^{b} \end{array} & \begin{array}{cc} 0 & \omega_{x}^{b}\\ {-\omega }_{x}^{b} & 0 \end{array} \end{array}\right],\mathrm{and}\;\psi \left(q\left(t\right)\right)=\left[\begin{array}{cc} \begin{array}{cc} q_{4} & -q_{1}\\ q_{1} & q_{4} \end{array} & \begin{array}{cc} {-q}_{2} & {-q}_{3}\\ {-q}_{3} & q_{2} \end{array}\\ \begin{array}{cc} q_{2} & q_{3}\\ q_{3} & -q_{2} \end{array} & \begin{array}{cc} q_{4} & {-q}_{1}\\ q_{1} & q_{4} \end{array} \end{array}\right]$.

Due to the lack of star sensitivity data, the star sensitivity prediction quaternion is used to replace the censored star sensitivity measurement data as follows:

(13) $$Z=\left\{\begin{array}{cc} q_{eout} & t\leq t_{\tau },t\geq t_{end}\\ \tilde{q} & t_{\tau }<t<t_{end} \end{array}\right.,$$

where Z denotes the actual measured data; $q_{eout}$ is the output measurement quaternion when the star sensitivity is normal; $t_{\tau }$ is the time when the star sensor data are missing; and $t_{end}$ is the time when the star sensor outputs attitude recovery.

## 3. Proposed Attitude Estimation Algorithm

### 3.1. Tobit Kalman Filter Algorithm Framework

In this section, a TKF method for short-time spacecraft attitude determination when the spacecraft is in a nearly circular orbit and star sensor data are missing is proposed. First, the gyro drift is estimated from the measured data of a star sensor in the period before the period where data are missing. Considering the influence of the amount of missing data, the model error is determined using statistical information on the censored data to minimize the covariance of the estimation error. Finally, the model error is compensated to obtain accurate attitude information.

The quaternion estimation error is defined as an algebraic error by $∆q=q-\tilde{q}$. ∆q is only a mathematical description [28], and the corrected quaternion is provided by:

(14) $$q\left(t\right)=\tilde{q}\left(t\right)+∆q\left(t\right).$$

The state prediction model represents the integration on the time interval of [t−Δt,t], and the prediction model of a system state is defined as follows:

(15) $$\tilde{q}\left(t\right)=q\left(t-\Delta t\right)+\frac{1}{2}\Omega \left(\omega^{b}\right)q\left(t-\Delta t\right)\cdot \Delta t,$$

(16) $$\tilde{b}\left(t\right)=b\left(t-∆t\right)+n_{d}\cdot \Delta t,$$

Substituting Equation (9) into Equation (12) yields:

(17) $$\dot{q}\left(t\right)=\frac{1}{2}\Omega \left(\omega^{b}\right)q\left(t\right)-\frac{1}{2}\psi \left(q\left(t\right)\right)\cdot b^{o}\left(t\right),$$

where $b^{o}\left(t\right)={\left[\begin{array}{cccc} 0 & b_{x} & b_{y} & b_{z} \end{array}\right]}^{T}$ is the extended gyro drift.

The difference between the angular velocity calculated from the star sensor measurement data and the gyro measurement result represents the gyro drift value, and it is expressed as follows:

(18) $$b^{o}\left(t\right)={\left[{\left(-\frac{1}{2}\psi \left(\tilde{q}\left(t\right)\right)∆t\right)}^{T}\left(-\frac{1}{2}\psi \left(\tilde{q}\left(t\right)\right)∆t\right)\right]}^{-1}{\cdot \left(-\frac{1}{2}\psi \left(\tilde{q}\left(t\right)\right)∆t\right)}^{T}\cdot \left[q\left(t+∆t\right)-\tilde{q}\left(t\right)-\frac{1}{2}\Omega \left(\omega^{b}\right)\tilde{q}\left(t\right)∆t\right].$$

Based on Equations (15)–(17), the state equation can be rewritten as follows:

(19) $$x_{k}=A\cdot x_{k-1}+w_{k-1},$$

(20) $$A=\left[\begin{array}{cc} I_{4\times 4}+\frac{1}{2}\Omega \left(\omega^{b}\right)\Delta t & -\frac{1}{2}\psi \left(q\right)\Delta t\\ 0_{4\times 4} & I_{4\times 4} \end{array}\right]$$

where $x={\left[\begin{array}{cc} \begin{array}{cccc} q_{4} & q_{1} & q_{2} & q_{3} \end{array} & \begin{array}{cccc} 0 & b_{x} & b_{y} & b_{z} \end{array} \end{array}\right]}^{T}$ is a state variable, and $w_{k}=\left[\begin{array}{c} n_{q}\left(t\right)\\ 0\\ n_{d}\Delta t \end{array}\right],$ $n_{q}\left(t\right)$ is the measurement error of the star sensor.

In the improved system, the gyroscope drift value is considered as the measured value. Considering the high-precision measurement performance of a star sensor, the prediction error is calculated using the star sensor measurement data at time (t+∆t) as reference data. Based on Equation (18), the latent measured value in Equation (1) can be rewritten as follows:

(21) $$y_{k}^{*}={\left[{\left(-\frac{1}{2}\psi \left(\tilde{q}\left(t\right)\right)∆t\right)}^{T}\left(-\frac{1}{2}\psi \left(\tilde{q}\left(t\right)\right)∆t\right)\right]}^{-1}{\left(-\frac{1}{2}\psi \left(\tilde{q}\left(t\right)\right)∆t\right)}^{T}\left[q\left(t+∆t\right)-\tilde{q}\left(t\right)-\frac{1}{2}\Omega \left(\omega^{b}\right)\tilde{q}\left(t\right)∆t\right]+v_{k},$$

where $y_{k}^{*}={\left[\begin{array}{cccc} 0 & b_{x} & b_{y} & b_{z} \end{array}\right]}^{T}$; $v_{k}$ is measurement noise; $E\left(v_{k}\right)=0;\;E\left(v_{k}{v_{k}}^{T}\right)=R$; and R is the measurement noise covariance matrix.

Moreover, τ is the measured value at the time of star sensor failure.

(22) $$\tau =y_{k}^{*}\left(t_{\tau }\right)$$

The system state estimation performance is affected by the statistics of truncation measurement data. Therefore, the main objective of the attitude determination TKF method is to consider the statistical information of censored measurement data fully to estimate the latent information accurately.

The TKF method under the integrated navigation system is as follows [14].

The prior estimation of a system state and its probability distribution can be, respectively, expressed as follows:

(23) $$P\left(x_{k\left|k-1\right.}\right)\sim N\left(E\left(x_{k\left|k-1\right.}\right),Var\left(x_{k\left|k-1\right.}\right)\right).$$

The prediction equation of the system state is defined by:

(24) $$E\left(x_{k\left|k-1\right.}\right)=Ax_{k-1\left|k-1\right.},$$

where $x_{k-1\left|k-1\right.}$ is the estimated value at time (k−1).

The covariance of the state error of a given measurement value and state information at time (k−1) is expressed by:

(25) $$cov\left(x_{k\left|k-1\right.}-x_{k}\right)=A\psi_{k-1\left|k-1\right.}A^{T}+Q,$$

where $\psi_{k-1\left|k-1\right.}$ is a prior estimation of the state error covariance, and Q is the system noise covariance matrix.

The optimal Kalman filter minimizes the state error covariance ($\psi_{k\left|k\right.}$). The following update steps minimize the state error covariance.

The estimation equation is

(26) $$x_{k\left|k\right.}=x_{k\left|k-1\right.}+R_{\tilde{x}{\tilde{y}}_{k}}R_{\tilde{y}{\tilde{y}}_{k}}^{-1}\left(y_{k}-E\left(y_{k}\right)\right).$$

where $R_{\tilde{x}{\tilde{y}}_{k}}$ is the cross-covariance between the measurement error and the current system state, and $R_{\tilde{y}{\tilde{y}}_{k}}$ is the variance of the measurement error; they are calculated by:

(27) $$R_{\tilde{x}{\tilde{y}}_{k}}=E\left(\left(x_{k}-x_{k\left|k-1\right.}\right){\left(y_{k}-E\left(y_{k}\right)\right)}^{T}\right),$$

(28) $$R_{\tilde{y}{\tilde{y}}_{k}}=E\left(\left(y_{k}-E\left(y_{k}\right)\right){\left(y_{k}-E\left(y_{k}\right)\right)}^{T}\right),$$

where $E\left(y_{k}\right)$ is calculated by Equation (7), and $E\left(y_{k}\right)\in R^{4*1}$.

According to Equation (26), it holds that:

(29) $$\psi_{k\left|k\right.}=cov\left(x_{k}-x_{k\left|k\right.}\right)=cov\left(x_{k}-x_{k\left|k-1\right.}-K_{k}\left(\mu_{k}\left(Cx_{k}+v_{k}\right)+\left(I_{4\times 4}-\mu_{k}\right)\tau -E\left(y_{k}\right)\right)\right),$$

and then the Kalman gain is computed by:

(30) $$K_{k}=R_{\tilde{x}{\tilde{y}}_{k}}R_{\tilde{y}{\tilde{y}}_{k}}^{-1}.$$

Next, calculate the measurement error by:

(31) $${\tilde{y}}_{k}=\mu_{k}\left(Cx_{k}+v_{k}\right)+\left(I_{4\times 4}-\mu_{k}\right)\tau -E\left(y_{k}\right).$$

Then, the covariance of the system state estimation is expressed by:

(32) $$\psi_{k\left|k\right.}=E\left(\left(x_{k}-x_{k\left|k-1\right.}-K_{k}{\tilde{y}}_{k}\right){\left(x_{k}-x_{k\left|k-1\right.}-K_{k}{\tilde{y}}_{k}\right)}^{T}\right)=\psi_{k\left|k-1\right.}-E\left(\left(x_{k}-x_{k\left|k-1\right.}\right){\tilde{y}}_{k}^{T}\right)K_{k}^{T}-K_{k}E\left({\tilde{y}}_{k}{\left(x_{k}-x_{k\left|k-1\right.}\right)}^{T}+K_{k}E\left({\tilde{y}}_{k}{\tilde{y}}_{k}^{T}\right)K_{k}^{T}\right),$$

the cross-covariance between the measurement error is computed by:

(33) $$R_{\tilde{x}{\tilde{y}}_{k}}=E\left(\left(x_{k}-x_{k\left|k-1\right.}\right){\left(\mu_{k}\left(y_{k}^{*}-Cx_{k}\right)+(I_{4\times 4}-\mu_{k})\tau -E(y_{k})\right)}^{T}\right).$$

The cumulative distribution of the measured data represents a function of the distance between the invisible measured variable and the threshold. The expectation of $\mu_{k}\left(l\right)$ is calculated by:

(34) $$E\left(\mu_{k}\left(l\right)\right)=\Phi \left(\frac{\tau \left(l\right)-Cx_{k}\left(l\right)}{\sigma \left(l\right)}\right),$$

where $Cx_{k}\left(l\right)$ is the *l*th element of the measurement vector, and σ(l) is the variance of the noise on this element.

For simplicity, the following assumptions are made.

**Assumption** **1.** *It is assumed that the quantity of the measured data is irrelevant; therefore, R is a diagonal matrix, and it holds that:*

(35) $$cov\left(y_{k}\left(d\right),y_{k}\left(l\right)\right)=0,\forall d\neq l.$$

**Assumption** **2.** *The state prediction represents a sufficient and accurate estimation of the censored data probability, and it is provided by:*

(36) $$E\left(\mu_{k}\left(l\right)\right)=\Phi \left(\frac{\tau \left(l\right)-Cx_{k}\left(l\right)}{\sigma \left(l\right)}\right)\approx \Phi \left(\frac{\tau \left(l\right)-Cx_{k\left|k-1\right.}\left(l\right)}{\sigma \left(l\right)}\right).$$

According to

(37) $$E\left(x_{k\left|k\right.}\right)=E\left(x_{k\left|k-1\right.}\right)=x_{k\left|k-1\right.},$$

and

(38) $$E\left(x_{k}x_{k}^{T}\right)=E\left(\left(x_{k}-E\left(x_{k\left|k-1\right.}\right)\right){\left(x_{k}-E\left(x_{k\left|k-1\right.}\right)\right)}^{T}\right)+E\left(x_{k}\right){E\left(x_{k}\right)}^{T}=\psi_{k\left|k-1\right.}+x_{k\left|k-1\right.}x_{k\left|k-1\right.}^{T},$$

obtain

(39) $$R_{\tilde{x}{\tilde{y}}_{k}}=\psi_{k\left|k-1\right.}C^{T}E\left(\mu_{k}\right).$$

For the same token, the variance of the measurement error is determined as:

(40) $$R_{\tilde{y}{\tilde{y}}_{k}}=E\left(\mu_{k}\right)C\psi_{k\left|k-1\right.}C^{T}E\left(\mu_{k}\right)+Var\left(y_{k}\left|x_{k\left|k-1\right.}\right.,\sigma \right).$$

Next, substitute the optimal Kalman gain into Equation (32) to obtain a simplified covariance update equation as follows:

(41) $$\psi_{k\left|k\right.}=\left(I_{4\times 4}-K_{k}E\left(\mu_{k}\right)C\right)\psi_{k\left|k-1\right.}.$$

When the state value is far away from the censored region, the TKF algorithm can be transformed into the standard Kalman filter [13]:

(42) $$lim\frac{Cx_{k\left|k-1\right.}-\tau }{\sigma }\to \infty \left\{\begin{array}{c} E\left(\mu_{k}\right)=I\\ E\left(y_{k}\right)=Cx_{k\left|k-1\right.}\\ R=\sigma^{2}\\ R_{\tilde{x}\tilde{y}}=C\psi_{k\left|k-1\right.}\\ R_{\tilde{y}\tilde{y}}=C\psi_{k\left|k-1\right.}C^{T}+R\\ \psi_{k\left|k\right.}=(I-K_{k}C)\psi_{k\left|k-1\right.} \end{array}.\right.$$

The TKF represents a generalized Kalman filter.

### 3.2. Improved Tobit Unscented Kalman Filter Algorithm

The TUKF is based on the traditional Kalman filter, using the sigma point set to calculate the expectation and variance after the unscented transformation (UT), and using the Tobit model to calculate the expectation and variance of relevant measured values in the attitude determination process.

First, the UT transformation is performed, and the corresponding weight value is calculated using [29]:

(43) $$\begin{array}{c} \omega_{0}^{m}=\frac{\lambda }{n+\lambda }\\ \omega_{0}^{c}=\frac{\lambda }{n+\lambda }+(1-\alpha^{2}+\beta )\\ \omega_{i}^{m}=\omega_{i}^{c}=\frac{1}{2(n+\lambda )}i=1,2,\ldots ,2n \end{array}.$$

where the superscript *m* is the mean, *c* is the covariance, the subscript is the number of sampling points, and $\lambda =\alpha^{2}\left(n+k\right)-n$.

Next, define a sigma point set as follows [29]:

(44) $$\begin{array}{c} x_{k-1}^{0}=x_{k-1\left|k-1\right.}\\ x_{k-1}^{i}=x_{k-1\left|k-1\right.}+\sqrt{\left(n+1\right)P_{k-1}},i=1,2,\ldots ,n\\ x_{k-1}^{i}=x_{k-1\left|k-1\right.}-\sqrt{\left(n+1\right)P_{x}},i=n+1,n+2,\ldots ,2n \end{array}.$$

Then, the state equation is transformed by the UT, the sigma points are substituted into the nonlinear state function, and the one-step predictive state value and predictive covariance matrix value are obtained by weighting.

The prediction equation of sigma point set is

(45) $$X_{k|k-1}^{i}=f\left(X_{k-1}^{i}\right),$$

where X represents the state value of the Sigma point set.

The predicted sigma point is provided by [29]:

(46) $$X_{k|k-1}=\sum_{i=0}^{2n}\omega_{m}^{i}X_{k/k-1}^{i}.$$

Further, the error covariance is calculated by:

(47) $$\psi_{k\left|k-1\right.}=\sum_{i=0}^{2n}\omega_{i}^{c}{(X}_{k/k-1}^{i}-X_{k|k-1}){{(X}_{k/k-1}^{i}-X_{k|k-1})}^{T}+Q.$$

The prediction equation of the sigma point is defined by:

(48) $$\gamma_{k/k-1}^{i}=CX_{k/k-1}^{i}.$$

The variance of the measurement error is provided by:

(49) $$R_{\tilde{y}{\tilde{y}}_{k}}=\sum_{i=0}^{2n}\omega_{i}^{c}(\gamma_{k/k-1}^{i}-E(y_{k})){{(\gamma }_{k/k-1}^{i}-E(y_{k}))}^{T}+Var\left(y_{k}\left|x_{k}\right.,\sigma \right).$$

The cross-covariance between the measurement error and the current system state is provided by:

(50) $$R_{\tilde{x}{\tilde{y}}_{k}}=\sum_{i=0}^{2n}\omega_{i}^{c}\left(X_{k/k-1}^{i}-{\hat{x}}_{k}^{-}\right){{(\gamma }_{k/k-1}^{i}-{\hat{y}}_{k}^{-})}^{T}.$$

The status values and covariance matrix values are updated as follows:

(51) $$K=R_{\tilde{x}{\tilde{y}}_{k}}{R_{\tilde{y}{\tilde{y}}_{k}}}^{-1},$$

(52) $$\psi_{k\left|k\right.}=\left(I_{4\times 4}-K_{k}E\left(\mu_{k}\right)C\right)\psi_{k\left|k-1\right.},$$

(53) $$x_{k\left|k\right.}=x_{k\left|k-1\right.}+K_{k}\left(y_{k}-E\left(y_{k}\right)\right).$$

## 4. Simulation

In this part, some simulations were carried out to compare the accuracy of the different algorithms, and the influence of the Tobit model on the estimated value was analyzed when the measured value was truncated.

### 4.1. Comparison between the Straight Kalman Filter and the Tobit Kalman Filter

**Figure 1:**
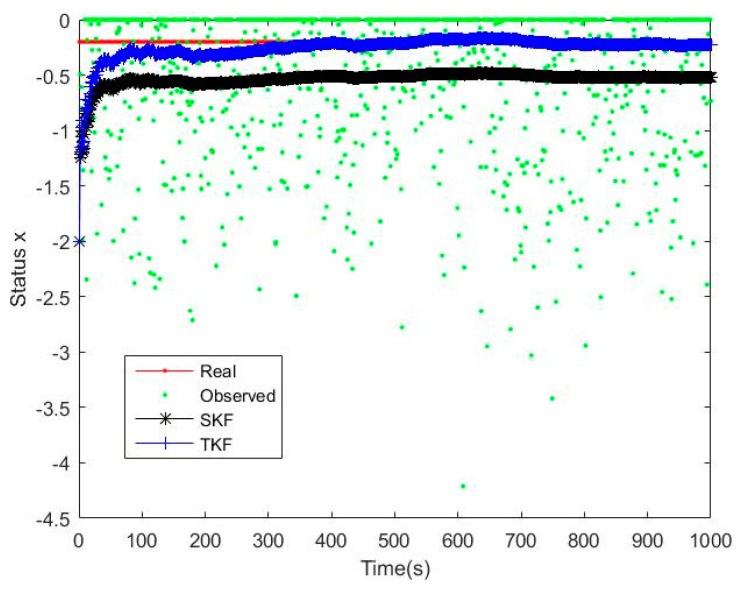
Filtering effect of SKF and TKF.

**Figure 2:**
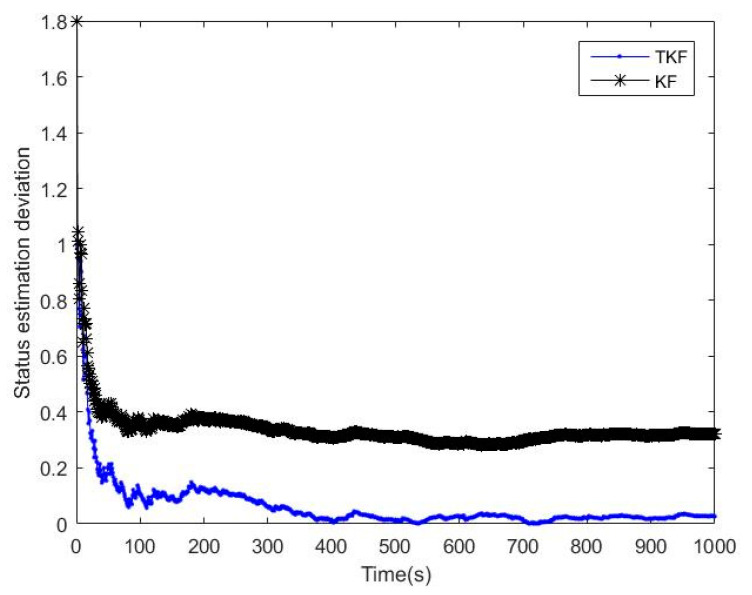
Filtering deviation between SKF and TKF.

The dynamic equation of the simulation system was shown in Equation (54), $w_{k-1}$ and $v_{k}$ were Gaussian white noise with a zero mean value, the variance were $Q=10^{-11},\;R=1$, the truncation threshold was τ=0, the initial values were set as $x_{0}=-0.2,P_{0}=1$, and the number of iterations was 1000, as shown in Figure 1 and Figure 2.

(54) $$\begin{array}{c} x_{k}=-0.2+w_{k-1}\\ y_{k}^{*}=x_{k}+v_{k}\\ y_{k}=\left\{\begin{array}{c} y_{k}^{*},y_{k}^{*}<\tau \\ \tau ,y_{k}^{*}\geq \tau \end{array}\right. \end{array}.$$

From the analysis results in Figure 1 and Figure 2, it was found that many of the observed data were set to 0 due to truncation, but TKF eventually converges to the true value, and the straight Kalman filter (SKF) [30] eventually had a nearly constant deviation from the true value. It proved that the Standard Kalman Filter is a biased estimate under the Tobit model, and the deviation was proved in Equation (7). TKF can successfully converge because it calculated the probability distribution of the observations under the Tobit model in advance and made corresponding corrections during the updating process of a linear Kalman filter, predicted the hidden information under the Tobit model, and used this information to complete the unbiased estimation.

### 4.2. Comparison between the Extended Kalman Filter and the Tobit Extended Kalman Filter

**Figure 3:**
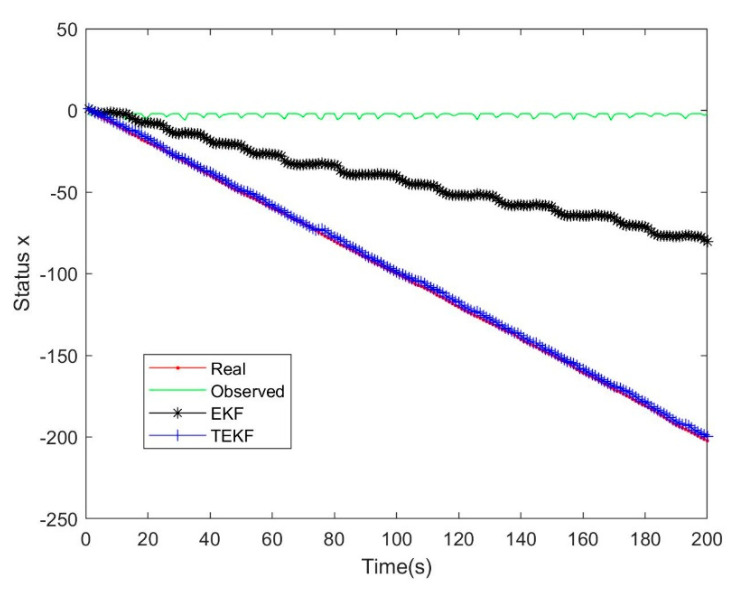
Filtering effect of EKF and TEKF.

**Figure 4:**
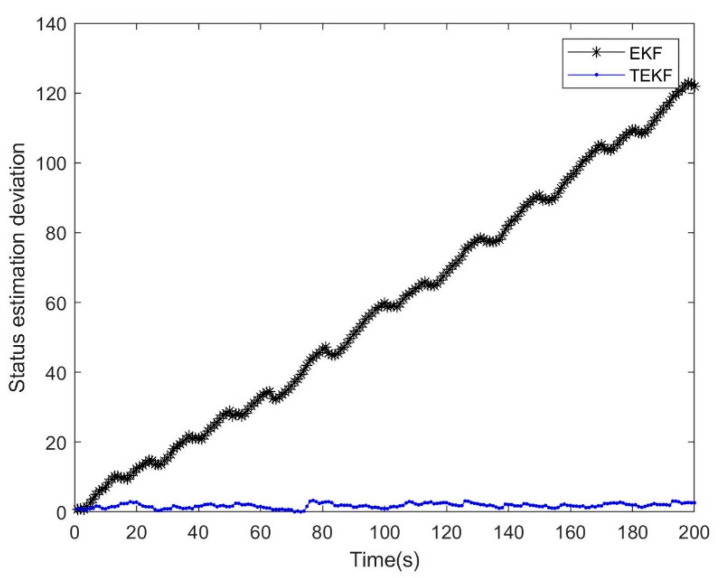
Filtering deviation between EKF and TEKF.

The Tobit extended Kalman filter (TEKF) is described in [13]. Due to space limitations in the article, it will not be repeated here. The dynamic equation of the simulation system was shown in Equation (55), $w_{k-1}$ and $v_{k}$ were Gaussian white noise with zero mean value, the variance were Q=0.04, R=1, the truncation threshold was τ=−2, the initial values were set as $x_{0}=0.2,\;P_{0}=1$, and the number of iterations was 200, as shown in Figure 3 and Figure 4.

(55) $$\begin{array}{c} x_{k}=x_{k-1}-1+w_{k-1}\\ y_{k}^{*}=-5cos\;(x_{k})+v_{k}\\ y_{k}=\left\{\begin{array}{c} y_{k}^{*},y_{k}^{*}<\tau \\ \tau ,y_{k}^{*}\geq \tau \end{array}\right. \end{array}.$$

The analysis of the results in Figure 3 shows that many of the observed data were set to −2 due to truncation, but, while the EKF results were divergent, the TEKF can still maintain a good tracking effect in the end. In terms of the results, the only difference between the two was the prior knowledge about the observed data. The TEKF recalculated the numerical characteristics of the observed values based on the Tobit model, while EKF was still the assumption of the Gaussian continuous distribution. Therefore, there were deviations in the working process and the deviations accumulate in the time-varying system, leading to divergence. In addition, after many tests, it was found that the robustness of EKF and TEKF was not good enough.

### 4.3. Comparison between the Unscented Kalman Filter and the Tobit Unscented Kalman Filter

**Figure 5:**
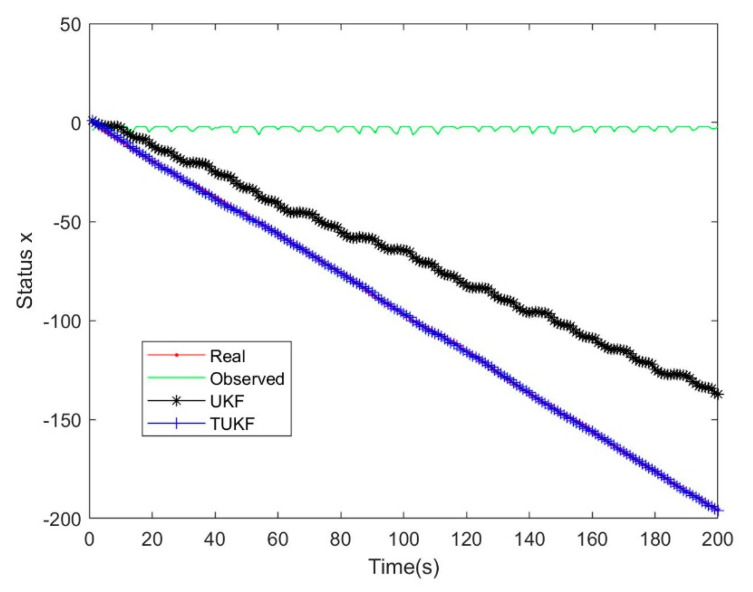
Filtering effect of UKF and TUKF.

**Figure 6:**
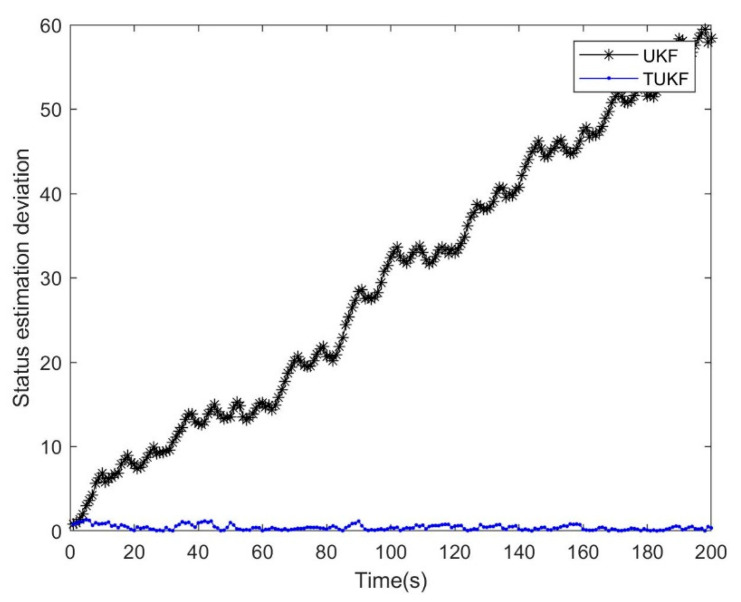
Filtering deviation between UKF and TUKF.

The simulation used the same system in Section 4.2, and the results are shown in Figure 5 and Figure 6.

In the process of the simulation experiments, the TUKF achieved good tracking results. Compared with the TEKF, the TUKF also achieved better results. The analysis showed that the higher error of the TEKF comes from the Taylor expansion, which only carries out first-order approximation and ignores the results of other orders.

### 4.4. Simulation Analysis of TUKF in Attitude Determination

**Figure 7:**
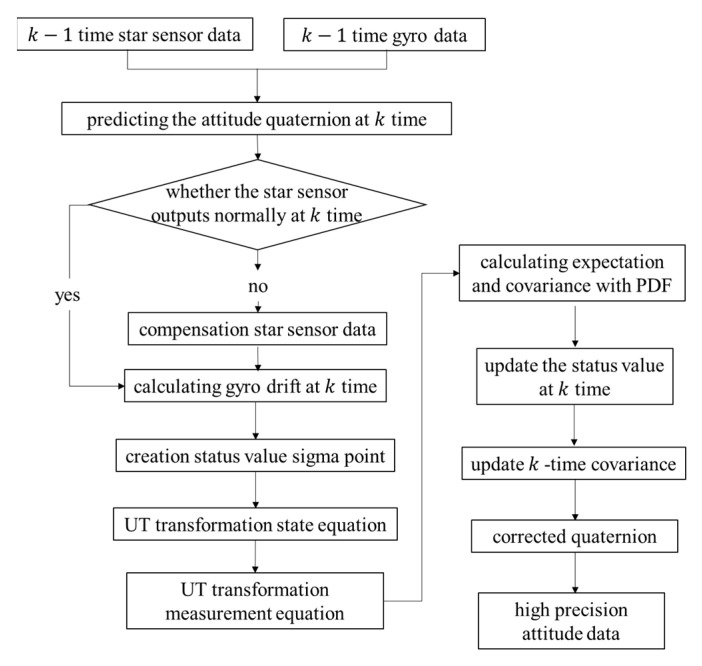
The flowchart of the TUKF algorithm.

The flow chart of the TUKF is shown in Figure 7.

The proposed design is verified via simulation experiments in the MATLAB software environment. The parameters were set as follows: the star sensor and gyro strap-down installation coordinates coincided; the gyro measurement white noise was set to $n_{1}=5deg/h\left(3\sigma \right)$; the initial value of the gyro drift was set to $d\left(0\right)={\left[0.5,0.5,0.5\right]}^{T}deg/h$; the standard deviation of white noise was $n_{d}=0.1deg/h(3\sigma )$; the gyro constant drift was $b_{0}=1deg/h(3\sigma );$ and the measurement noise of a star sensor was s=1″(3σ). The initial attitude angle was $\varphi_{0}={\left[-40,5,-34\right]}^{T}deg$; the initial value of the three-axis angular velocity was $\omega_{0}={\left[0.05,0.0003,0.02\right]}^{T}deg/s$; and the star sensor output frequency was 10 Hz, simulation duration was 2000 s, star sensor attitude failure data truncation occurred at 400 s, and the measured value at this truncation time was the truncation threshold τ.

#### 4.4.1. Measurement Error Covariance Matrix Effect on Filtering Performance

The assumption is that the measurement error covariance information was known before, but, in this process, the measurement error fluctuation changed when the star sensor failed. Set the duration of the star sensor failure to 15 min. Then, the measurement error covariance was assumed artificially, and three conditions of $R={n_{1}}^{2}\cdot I_{4\times 4}$, $R={n_{1}}^{2}{\cdot 10}^{-2}{\cdot I}_{4\times 4}$, and $R={n_{1}}^{2}{\cdot 10}^{-4}\cdot I_{4\times 4}$ were analyzed.

**Figure 8:**
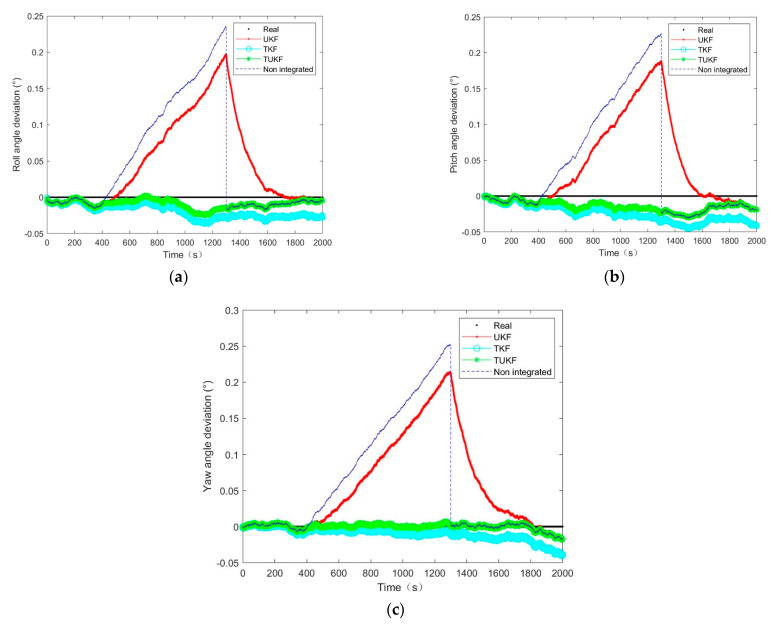
The attitude angle estimation error at a different measurement error covariance $R={n_{1}}^{2}\cdot I_{4\times 4}$: (**a**) roll angle; (**b**) pitch angle; (**c**) yaw angle.

**Figure 9:**
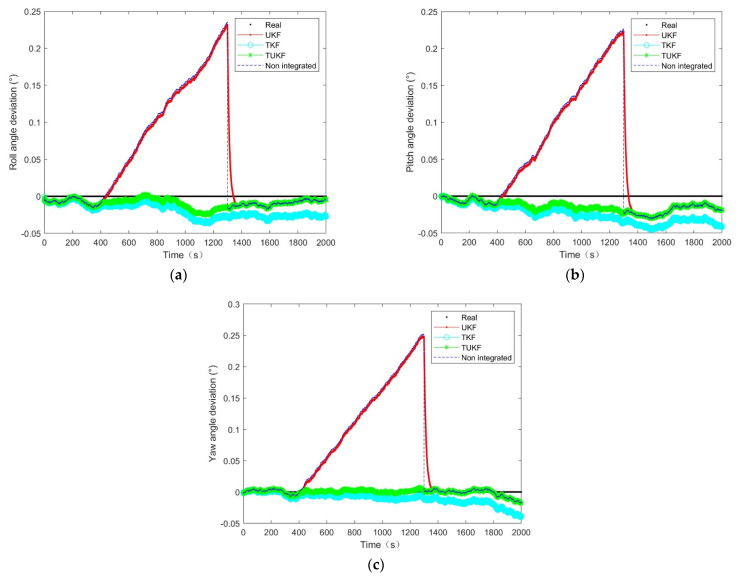
The attitude angle estimation error at a different measurement error covariance $R={n_{1}}^{2}{\cdot 10}^{-2}{\cdot I}_{4\times 4}$: (**a**) roll angle; (**b**) pitch angle; (**c**) yaw angle.

**Figure 10:**
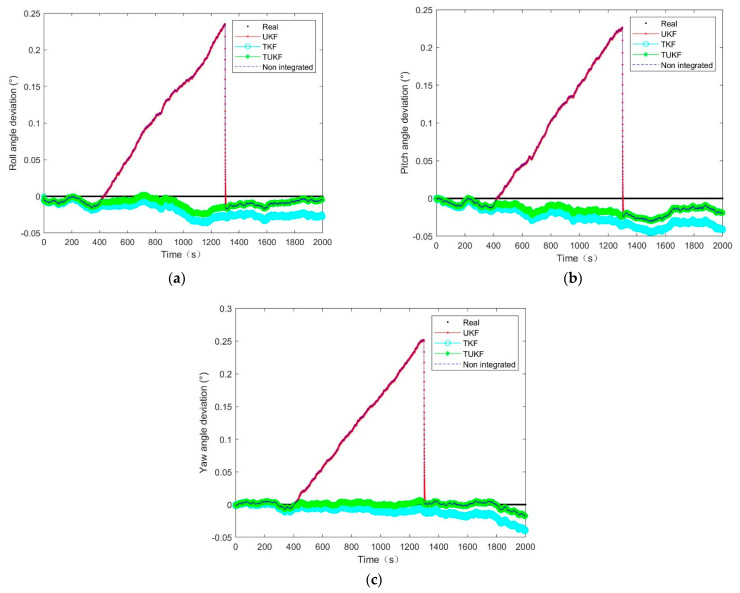
The attitude angle estimation error at a different measurement error covariance $R={n_{1}}^{2}{\cdot 10}^{-4}\cdot I_{4\times 4}$: (**a**) roll angle; (**b**) pitch angle; (**c**) yaw angle.

In this system, a combination of star sensors and gyroscopes was considered to determine the attitude. The term ‘no integration’ in Figure 8, Figure 9 and Figure 10 refers to using a gyroscope to determine attitude in the truncated region and using the traditional star sensors and gyroscope combinations to determine the attitude at other times. The star sensor was available at 0–400 s and 1300–2000 s while the values at other times were truncated and the attitude determination ability was affected.

The simulation results of the 15 min star sensor attitude loss showed that, after the star sensor data loss, the equivalent measurement error covariance was close to the actual situation, and the UKF error slowly diverged over time, as shown in Figure 8. When the covariance of the equivalent measurement error deviated from the actual situation, the filtering effect of the UKF degraded and was equivalent to the error caused by the gyro drift, as shown in Figure 9 and Figure 10. After the star sensor was output normally following the star sensor data being regressed, the closer the measurement error covariance was to the actual situation, the shorter the UKF error convergence time was, as shown in Figure 9 and Figure 10. Further, it can be seen in Figure 8 that, when the covariance of the equivalent measurement error deviated from the actual situation, the UKF filtering effect converged slowly. In contrast, the TKF and TUKF based on the Tobit model could accurately estimate the measurement value through probability statistics calculation and estimate the measurement error covariance in real time, showing a higher filtering estimation accuracy and stronger robustness than UKF.

Further, according to $\sigma k=\sqrt{\frac{\sum_{i=1}^{n}{\left(y_{ik}\right)}^{2}}{n}},where\;k=1,2,3$, n is the number of output quantities and the estimated attitude obtained by 50 operations. The mean square error results of the attitude obtained via different algorithms during the period of missing star sensor data under different measurement error covariance values are presented in Table 1. Compared with the UKF algorithm, the accuracy of the TUKF algorithm was improved by approximately 90% when the star sensitivity data were missing, which indicated the high filtering estimation accuracy of the proposed algorithm based on the Tobit model and confirmed the above results.

Figure 8, Figure 9 and Figure 10 clearly indicated that the errors of the TKF and the TUKF were relatively stable. At the truncation moment, the UKF error increased with the gyro drift, and the TKF and the TUKF can almost accurately tracked the spacecraft’s attitude. Table 1 shows that, under different measurement error covariance differences, the attitude estimation error of the TUKF was 60″, and the TKF was 80″ in the truncated region. This filter had a certain degree of robustness, meaning it was considered stable.

#### 4.4.2. Star Sensor Data Missing Duration Effect on Filtering Performance

**Figure 11:**
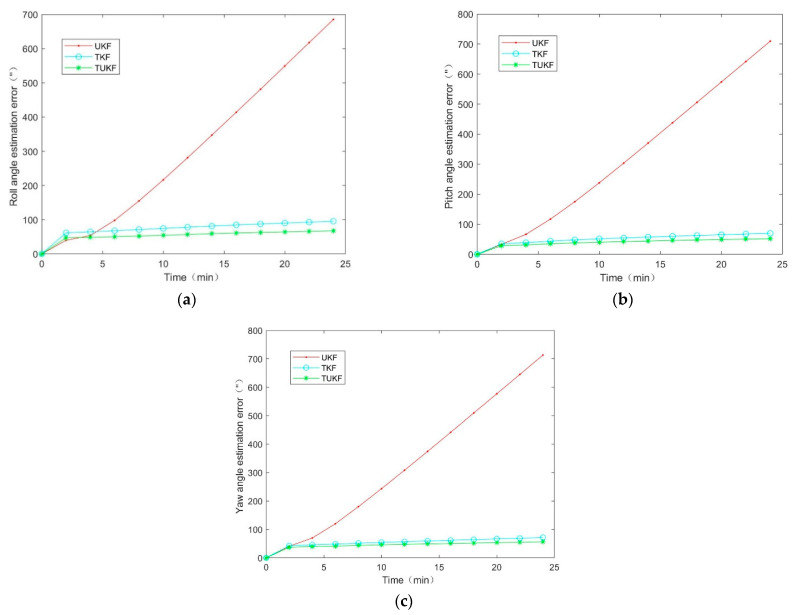
The estimation error results of the star sensor data for different failure times: (**a**) roll angle; (**b**) pitch angle; (**c**) yaw angle.

The failure duration of a star sensor is uncertain during the orbit operation of spacecraft. The measurement error covariance was $R={n_{1}}^{2}\cdot I_{4\times 4}$. According to the missing star sensor data of different durations, the mean square error results of the integrated navigation attitude in the missing period of the star sensor data obtained by 50 operations were simulated, and the results were shown in Figure 11.

The simulation results show that: the failure time of the star sensor was 0–24 min, when the star sensor failed for 2 min, the mean attitude estimation error $\sigma_{t=2\min}$ is ${\sigma ukf}_{t=2\min}=\left[39.9\;33.2\;40.7\right],\;{\sigma tkf}_{t=2\min}=\left[61.7\;35.1\;42.6\right],{\sigma tukf}_{t=2\min}=\left[47.1\;28.5\;37.3\right]$; when the star sensor failed for 24 min, the mean attitude estimation error $\sigma_{t=24\min}$ is ${\sigma ukf}_{t=24\min}=\left[685.5\;709.6\;713.11\right],\;{\sigma tkf}_{t=24\min}=\left[95.45\;69.83\;71.7\right],\;{\sigma tukf}_{t=24\min}=\left[67.8\;51.7\;56.6\right]$. During the period of star sensor failure, the mean square error of the spacecraft attitude estimation for TKF and TUKF was relatively stable compared to the UKF, and it was believed that the TKF and the TUKF was convergent. Further, as presented in Figure 11, the filtering estimation accuracy of the TUKF algorithm was higher than that of the TKF algorithm. This was because the TUKF algorithm performed the UT transformation near the estimated points to obtain the mean and covariance of the obtained sigma point sets and then directly mapped the sigma point sets, using the statistical information of the truncated measurement to approximate the state PDF; thus, the estimated attitude was closer to the true value than the TKF algorithm. The simulation results verified the effectiveness of the above methods and had certain engineering applications.

Consequently, when the time of the star sensor fails is less than 2 min, the traditional combined attitude determination algorithm UKF can be used for attitude estimation; when the time of the star sensor fails is longer than 2 min, the TUKF algorithm has higher filtering estimation accuracy and stronger robustness. However, in the real situation, there are few cases where the star sensor fails is longer than 24 min, and the more information missing, the more parameters need to be considered. Therefore, the problem of combined attitude determination with long-time star sensor data missing needs to be further discussed.

## 5. Conclusions

This paper proposes the TUKF based on the star sensor and inertial gyro combination. Aiming at the attitude output in the period before the star sensor becomes invalid, the missing information is estimated based on probability statistics using historical measurement data to overcome the difficulty of missing star sensor data. The proposed algorithm can accurately estimate the latent measurement values using the probability statistics calculation and the measurement error covariance in real time, showing a small performance loss compared to the UKF, the TKF, and using only gyro estimation. Therefore, the proposed TUKF algorithm can control the error between the estimated curve of the spacecraft attitude parameters and the true value of the spacecraft attitude in a short time under the condition of a short-term data loss, thus ensuring good real-time attitude determination effect of the system on the orbiting spacecraft, provided that there is theoretical support for the engineering practice. In this method, it is assumed that the prior information of the gyro drift is known; however, many factors affect gyro drift in actual orbit operation, and then affect the accuracy of the attitude determination. Therefore, further follow-up research on the calculation of gyro drift is required.

## Figures and Tables

**Table 1:** The mean square error results for different measurement error matrices. (″).

Measurement Error Matrix	Method	Roll	Pitch	Yaw
$25\cdot I_{4\times 4}$	UKF	380.71	404.13	408.22
	TKF	83.14	58.76	60.61
	TUKF	60.13	45.15	49.90
$25{\cdot 10}^{-2}\cdot I_{4\times 4}$	UKF	479.00	504.06	508.12
	TKF	83.11	58.75	60.67
	TUKF	60.13	45.14	49.89
$25{\cdot 10}^{-4}\cdot I_{4\times 4}$	UKF	489.53	514.70	518.85
	TKF	83.10	58.75	60.54
	TUKF	60.13	45.15	49.90

## Data Availability

Data to support the conclusion of the manuscript are included in the manuscript. Additional data are available upon reasonable request to the corresponding author.
